# Scientometric Research on Trend Analysis of Nano-Based Sustained Drug Release Systems for Wound Healing

**DOI:** 10.3390/pharmaceutics15041168

**Published:** 2023-04-06

**Authors:** Kuangyun Tang, Zhengyu Cai, Yanhan Lv, Ruiqi Liu, Qianming Chen, Jun Gu

**Affiliations:** 1 Stomatology Hospital, School of Stomatology, Zhejiang University School of Medicine, Zhejiang Provincial Clinical Research Center for Oral Diseases, Key Laboratory of Oral Biomedical Research of Zhejiang Province, Cancer Center of Zhejiang University, Hangzhou 310000, China; tangkuangyun@126.com (K.T.);; 2 Department of Burn and Plastic Surgery, West China Hospital, Sichuan University, Chengdu 610065, China; 3 Department of Cardiovascular Surgery, West China Hospital, Sichuan University, Chengdu 610065, China

**Keywords:** wound healing, nanocarrier, drug delivery, nanomedicine

## Abstract

Nanomaterials, such as the nanoparticle (NP), nanomicelle, nanoscaffold, and nano-hydrogel, have been researched as nanocarriers for drug delivery more and more recently. Nano-based drug sustained release systems (NDSRSs) have been used in many medical fields, especially wound healing. However, as we know, no scientometric analysis has been seen on applying NDSRSs in wound healing, which could be of great importance to the relevant researchers. This study collected publications from 1999 to 2022 related to NDSRSs in wound healing from the Web of Science Core Collection (WOSCC) database. We employed scientometric methods to comprehensively analyze the dataset from different perspectives using CiteSpace, VOSviewer, and Bibliometrix. The results indicated that China published the most significant number of documents in the last two decades, Islamic Azad Univ was the most productive institution, and Jayakumar, R was the most influential author. Regarding the analysis of keywords, trend topics indicate that “antibacterial”, “chitosan (CS)”, “scaffold”, “hydrogel”, “silver nanoparticle”, and “growth factors (GFs)” are the hot topics in recent years. We anticipate that our work will provide a comprehensive overview of research in this field and help scholars better understand the research hotspots and frontiers in this area, thus inspiring further explorations in the future.

## 1. Introduction

Skin is the largest organ in the human body, leading to skin wounds caused by various injuries and widespread damage. Skin wound repair is a complicated process in which new and healthy cells replace damaged cells, and damaged tissue structures are repaired. Wound healing involves a succession of sophisticated and orderly biological processes after tissue damage and defects, where local tissue is repaired by regeneration, repair, and reconstruction [1]. The delay of wound healing is strongly associated with infection, foreign bodies, local blood circulation disorders, and underlying diseases. Accordingly, a series of sustained-release drug delivery systems related to wound healing have been developed, such as hydrogel, microsphere, and scaffold systems [2,3,4]. In addition, drug delivery systems loaded with antibacterial drugs, growth factors, nucleic acid drugs, and active cells are applied through drug dissolution, chemical bond-controlled release, and material-controlled release to promote wound healing [5,6].

Nano-based drug sustained release systems (NDSRSs) combine nanotechnology and biomedicine. There are multiple advantages to applying nanotechnology in drug delivery system research. A sustained release system for drug delivery based on nanotechnology can achieve controllable drug release, improve drug penetration effectiveness, and protect bioactive substances, such as growth factors (GFs), DNA, RNA, and stem cells, from being quickly inactivated in vivo environments. With the NDSRSs, tissue regeneration and repair of wounds could be promoted by sterilization, inflammation control, reducing oxidative stress, and active substance release [7].

In the last 20 years, there have been many studies on nanotechnology-related drug delivery systems [8]. Researchers can accurately grasp the frontier of study and predict future research hotspots with the help of scientometric analysis. However, there has been no scientometric analysis of the research progress of nanotechnology-related drug delivery systems in wound healing. A comprehensive and real-time scientometric study will help scholars learn about the frontier of sustained drug release systems based on nanotechnology in wound healing, grasp the research direction, be familiar with the published studies, and understand the cooperation between different countries and institutions and authors. Consequently, our study aims to summarize the current research status of NDSRSsin wound healing. Meanwhile, we would like to discuss the hotspots of present research and future directions in this field.

## 2. Methods

### 2.1. Data Source and Search Strategy

**Figure 1:**
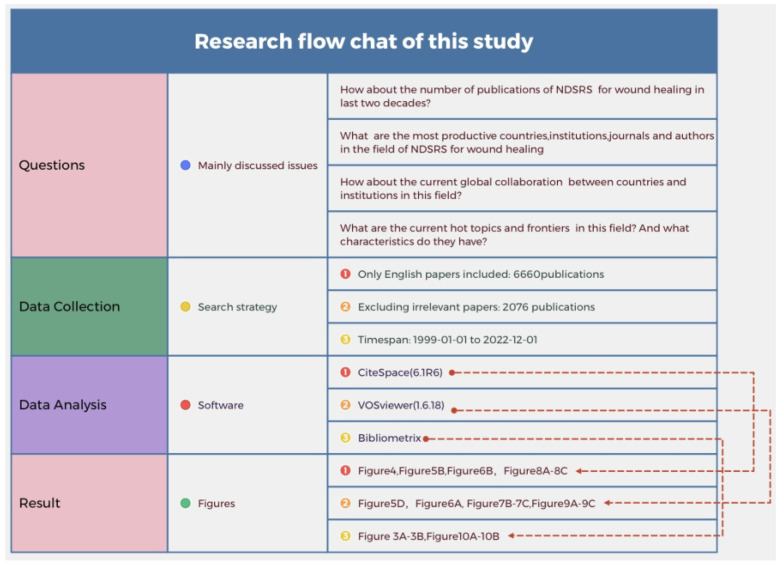
The flow chart of the scientometric study.

We comprehensively searched online publications in the WOSCC database on 30 December 2022. The retrieval strategy of this study is shown in Figure 1 and Supplementary S1 Detailed search strategy. The period of publication was from 1999 to 2022. To avoid bias caused by daily database updates, we searched articles on a single day. The language of publications was English; only “articles” and “reviews” were included. Before evaluating the article’s title and abstract to determine if the literature met the theme of NDSRSs in wound healing, we excluded publications that did not meet language and article type requirements.

### 2.2. Scientometric Analysis

Cite Space 6.1. R6 (Drexel University, USA) was used to analyze the included papers, including dual-map overlay of journals, the network map of countries and institutions, co-cited references, reference co-citation clusters and references with the strongest citation burst, and keywords with the strongest citation burst.

VOS viewer 1.6.18 (Leiden University, Holland) created the cooperation network of countries, institutions, authors, and co-cited authors and journals. A cluster analysis was conducted, and the VOS viewer created a network map and a density map for high-frequency keywords. In the visual map, different nodes represented countries, institutions, authors, etc. The size of the node was on behalf of the frequency or numbers. The color of the node and the line distinguished different clusters. At last, the thickness of the line reflected the strength of the link.

R-Bibliometrix (University of Naples Federico, Italy) was used to analyze the evolution trend of the topic over time according to the keywords, create a network map visualizing the global distribution of countries, and conduct a descriptive analysis of the publishing characteristics of journals. In addition, the scientometric online platform (https://bibliometric.com/) was also used to show the cooperation networks among countries. 

## 3. Results

### 3.1. Publication Outputs

**Figure 2:**
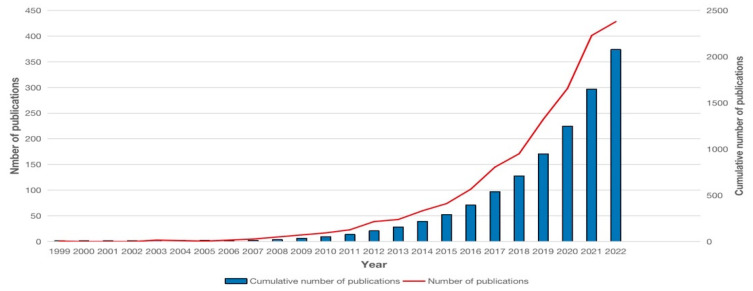
Publication years of nano-drug sustained release system research in wound healing.

We obtained 2076 articles published in English in total. The article by Hamouda, T in 1999 [9] is the first study on NDSRSsin wound healing, and the annual number of papers was under five before 2007 (Figure 2). From 2007 to 2015, the number of articles increased mildly, yet the academic output per year was still less than 100. After 2015, the number of publications each year overgrew, especially after 2016. The annually published papers gradually increased from 102 in 2016 to 428 in 2022. Between 2015 and 2022, 1783 studies were published, accounting for 85.89% of all papers.

### 3.2. Journals and Co-Cited Journals

**Figure 3:**
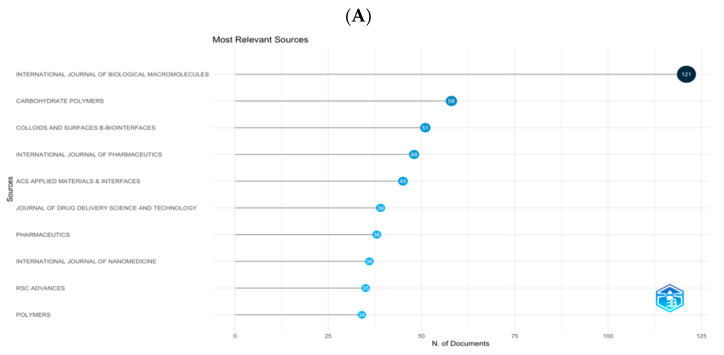

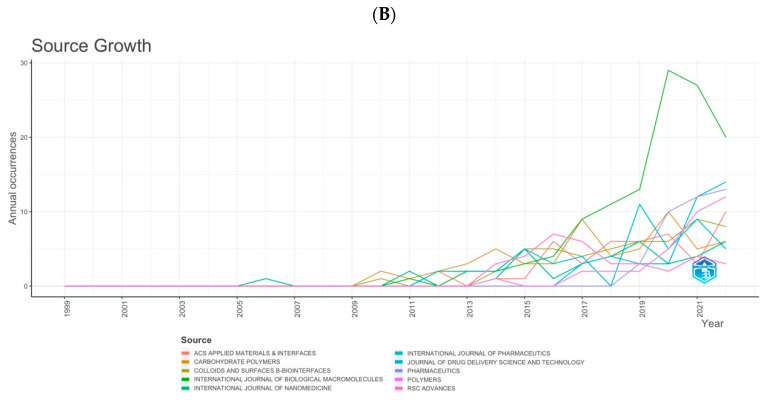
(**A**) The top 10 journals with the most publications generated by R-Bib liometrix. (**B**) Annual occurrences of the top 10 journals with the most publications generated by R-Bibliometrix.

Journals often cited together by other scholars are called co-cited journals. Four hundred eighty-seven journals published 2076 papers. Information on the top 10 and co-cited journals are presented in Table 1. As shown in Figure 3A, the top 10 journals which published 530 (24.37%) papers in total, INT J BIOL MACROMOL (121, 5.83%) ranked first, followed by CARBOHYD POLYM (59, 2.84%), COLLOID SURFACE B (51, 2.46%), INT J PHARMACEUT (48, 2.31%), and ACS APPL MATER INTER (45, 2.16%). Among the ten journals, four were from the Netherlands, two were from the United Kingdom (UK), two were from Switzerland, one was from France, and the last was from the USA. The average impact factors of the four journals were lower than 6.0. Figure 3B reflects the dynamic change in the annual publications of the most productive ten journals. It can be seen that from 2017 to 2020, publications of INT J BIOL MACROMOL increased rapidly, and this journal led the way in research in the last five years. As shown in Table 1, the top 10 co-cited journals’ co-citations were more than 1500. Six of these ten journals were published in the Netherlands, and 6 had impact factors higher than 10.0. The top 5 co-cited journals were CARBOHYDR POLYM (4560 co-citations), BIOMATERIALS (4113 co-citations), INT J BIOL MACROMOL (3575 co-citations), ACS APPL MATER INTER (2587 co-citations), and MAT SCI ENG C-MATER (2350 co-citations). Figure S1 shows the network visualization diagram of the journal co-citation analysis created by the VOS viewer. Seventy-five journals cited more than 300 times were included in the map.

**Figure 4:**
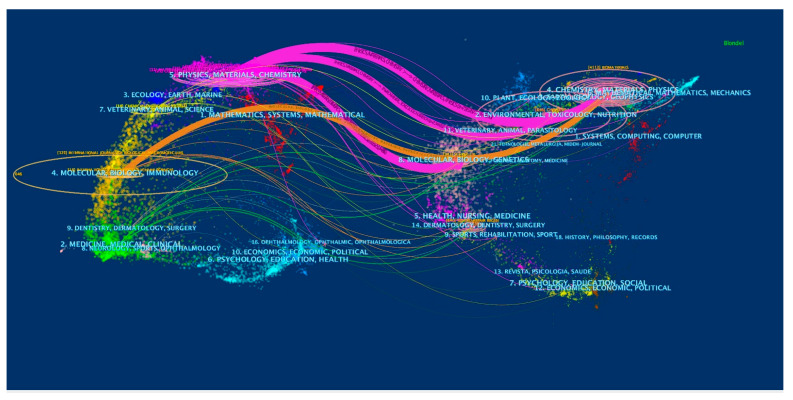
The dual-map overlay of journals related to NDSRSsin the wound healing field.

The dual-map overlay of journals revealed the overall scientific contribution. As shown in Figure 4, the left side represents the map of citing journals, and the right side represents the map of the cited journals. The label describes the subject covered by the journal. Colored line paths represent citation relationships. All paths originate from the citing map and point to the cited map, indicating the citation tracks of knowledge and knowledge flow. For example, four main citation paths were shown in the current map, suggesting that the citing papers concerning NDSRSsin wound healing primarily focused on journals in molecular biology, immunology physics, materials, and chemistry. At the same time, most of the cited articles were published in environmental science, toxicology, nutrition, chemistry, materials, and physics journals.

### 3.3. Countries and Institutions

**Figure 5:**
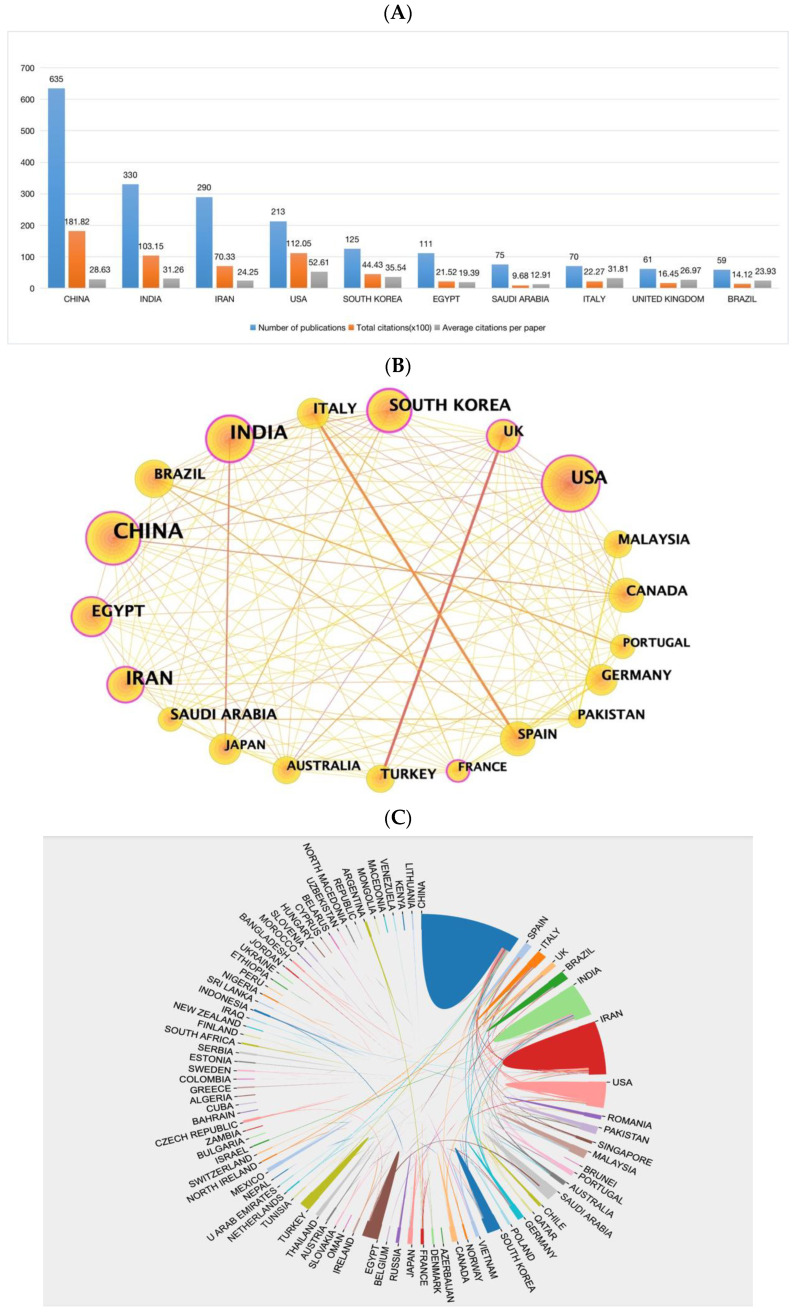

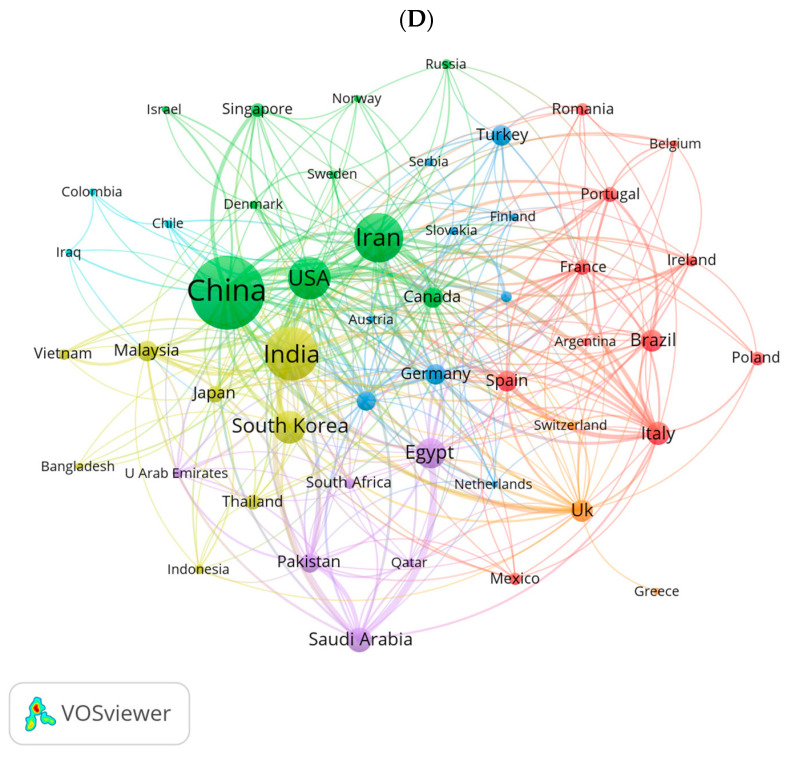
(**A**) The number of publications, total citations (×100), average citation per paper, and H-index of the 10 most productive countries/regions. (**B**) The country cooperation network of the top 20 productive countries generated by CiteSpace. (**C**) The international cooperation networks between countries. Line thickness between countries reflects the intensity of the closeness. (**D**) The network map for countries created by the VOS viewer.

A total of 89 countries contributed to the papers included. As shown in Table 2 and Figure 5A, China ranked first in the number of publications (n = 635, accounting for 30.59%), followed by India (n = 330, accounting for 15.90%; 10,315 citations) and Iran (n = 290, accounting for 13.97%). As to the number of total citations and average citations, it can be seen that China had the most significant number of total citations (n = 18182), followed by the USA (n = 11205), which ranked first among the top 10 countries in the average number of citations (with an average of 52.61 citations per paper), much higher than India and China (with the average of 31.26 and 28.63 citations respectively). Figure 5B,C show the communication and cooperation among countries. Figure 5B generated by CiteSpace, reveals the network map of the top 20 productive countries in this research area: The node size can reflect the number of publications proportionally, and the strength of cooperation between countries can be seen in the thickness of the direct connections between nodes. We found that in the top 20 countries, the United States, India, and China were the core countries of the network map, which had much more extensive and close cooperation with other countries. Some European countries (e.g., France, the UK, and Italy) and Asian countries (e.g., South Korea, Japan, and Iran) were also critical to the national cooperation map. Accordingly, it can be indicated that more extensive and close cooperative relations have been established between the developed countries.

In contrast, the communication between them and developing countries and within developing countries was still weak. In addition, the significance of nodes in the collaborative network can also be reflected by the betweenness centrality (BC) value. The purple outer circles highlight 7 nodes with BC values greater than 0.1, meaning they are essential nodes, including China, the USA, India, Iran, South Korea, Egypt, the UK, and France. Figure 5D was a network map for countries with publications over five conducted by VOS viewers. There were 50 nodes and 335 links on the map, which formed seven different clusters, and nodes distributed in the same cluster cooperated more closely. We also created a map (Figure S2) of the global distribution of these countries in research on NDSRSs for wound healing. The thickness of links between the two countries reflects the strength of cooperation, which indicates extensive cooperation between these countries. Many Asian countries like India, South Korea, and Japan cooperate closely with each other. China has the closest collaboration with the United States, Canada, and Iran, constituting the most significant transcontinental network. European countries like the UK, Italy, Spain, and Turkey also collaborate strongly. However, they do not have much collaboration and communication with Asian countries.

**Figure 6:**
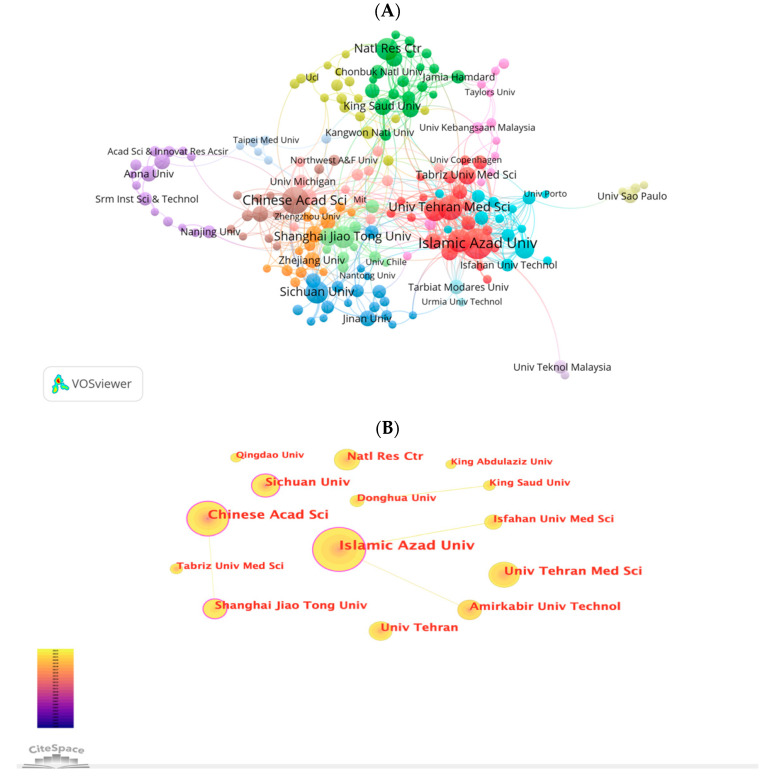
(**A**) The network map of institutions for NDSRSs in the wound healing field was created by VOSviewer. (**B**) The cooperation network of the top 20 institutions generated by Citespace.

A total of 2348 institutions contributed worldwide to the 2076 papers. The top 10 institutions contributed 380 (18.30%) articles (Table 2). Among the top 10 institutions, Islamic Azad University (67, 3.23%) published the highest number of articles, followed by Chinese Acad Sci (53, 2.55%), Univ Tehran Med Sci (41, 1.97%), Amirkabir University Technol (37, 1.78%) and Natl Res Ctr (38, 1.77%). VOS viewer generated a cooperation network of institutions, as shown in Figure 6A. 194 nodes and 690 links were in the network map of institutions with a collaborative frequency of greater than 5. The 194 institutions formed 15 clusters. Within the same cluster, the collaborations between institutions were especially active. Using Cite Space, we also created a cooperation network of the top 14 institutions. As shown in Figure 6B, institutions with BC values, greater than 0.1 were included, such as Islamic Azad University Chinese Acad Sci, Sichuan Univ, and Shanghai Jiao Tong Univ. However, there was little cooperation between them.

### 3.4. Authors and Co-Cited Authors

**Figure 7:**
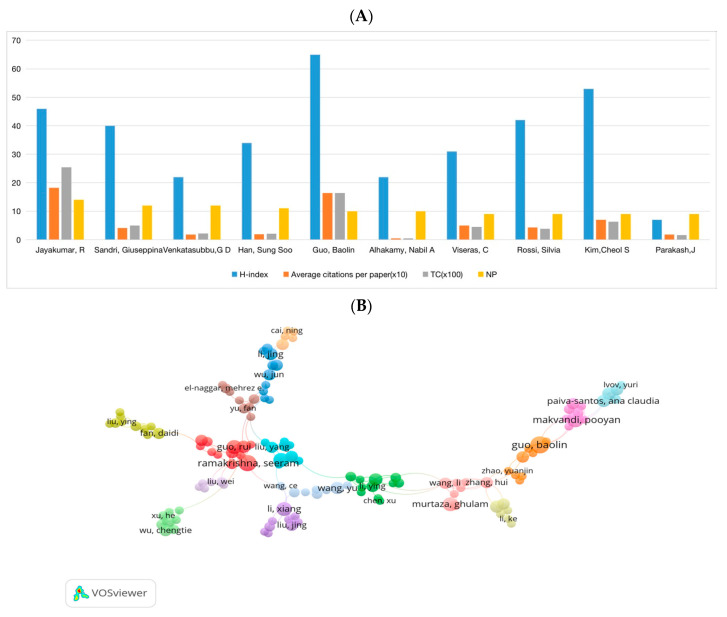

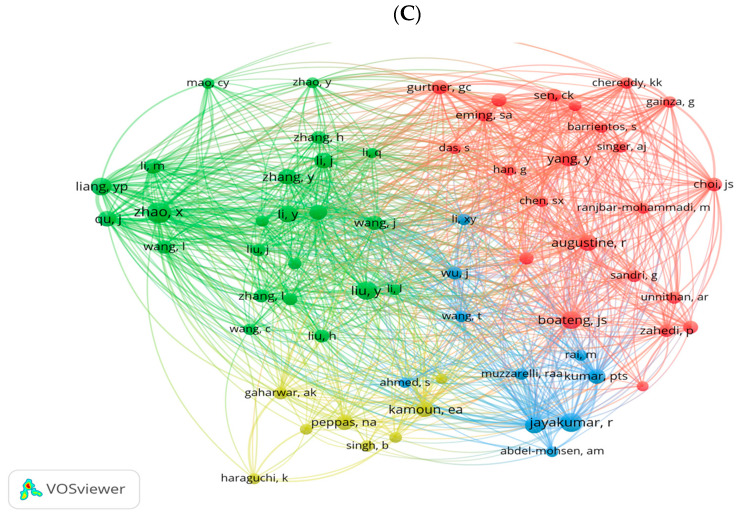
(**A**) The total citations (×100), average citation per paper (×10), and H-index of the top 5 most prolific authors. (**B**) The network map of authors for NDSRSs in the wound healing field. (**C**) Author co-citation analysis by VOSviewer.

This study created network maps of authors and co-cited authors using VOSviewer to provide information about influential research groups and authors. We obtained 10,579 authors in total from the 2076 papers. Table 3 shows the top 10 authors and co-cited authors. Concerning the analysis of authors, the total citations (×100), average citations per paper (×10), and H-index (an author-level metric that measures both the productivity and citation impact of the publications) of the top ten most prolific authors are shown in Figure 7A. These ten most productive authors published 83 (4.00%) papers. Among them, Jayakumar, R (14, 0.67%) contributed the most publications, followed by Sandri, Giuseppina (12, 0.59%), and Venkatasubbu, G D (12, 0.59%). The total citations of the papers by Jayakumar, R ranked first (n = 2545), with an average of 18.18 citations per paper (ranking first, too), and his H-index is 46. Although Guo, BL ranked second in total citations (n = 1640) and average citations per paper (n = 16.40), had an H-index of 65, much higher than other top ten productive authors. And nine of them had an H-index of above 20. Figure 7B shows the network map of authors who published more than three papers. It was found that the collaboration among these authors was not very close. As shown in Figure 7C, a co-citation network map of authors was conducted using VOSviewer. We defined 64 authors with more than 60 citations as influential researchers. The node size represents the citation frequency. The connection lines represent the cooperation between authors, and the thickness of the line represents the connection’s strength. The influence of an author’s published articles on other scholars within this research area can be reflected by total link strength (TLS). It can be seen that Zhao, X had the greatest TLS (n = 1408), followed by Liang, YP (n = 1084), and Qu, J (n = 910).

### 3.5. Co-Cited References and the Citation Bursts

**Figure 8:**
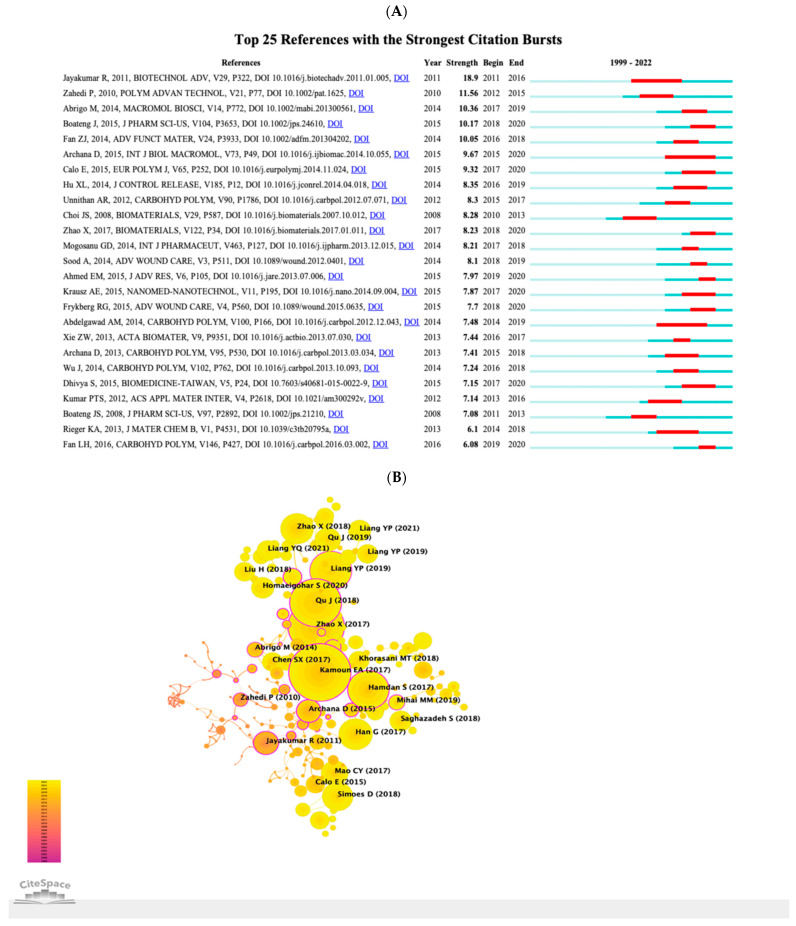

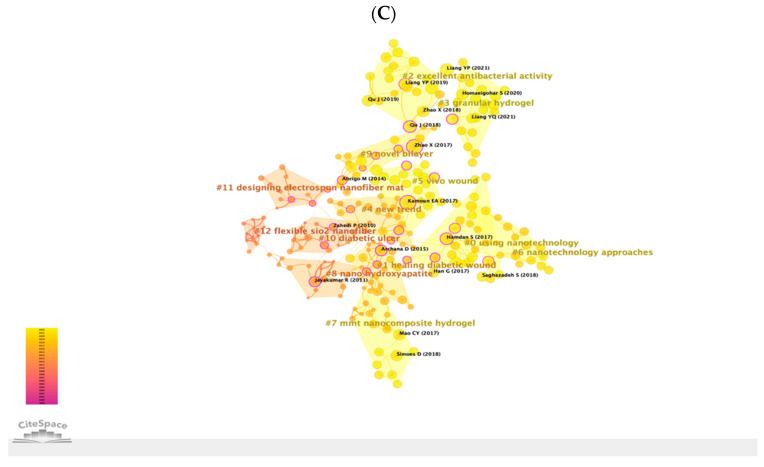
(**A**) The top 25 references with the strongest citation bursts in the co-citation network [10,11,19,20,21,22,23,24,25,26,27,28,29,30,31,32,33,34,35,36,37,38,39,40,41]. (**B**) The network map of reference co-citation for NDSRSs in the wound healing field. (**C**) The network map of reference co-citation clusters for NDSRSs in the wound healing field.

References cited by a series of articles are determined as co-cited references. Table 4 shows the ten references with the most co-citations related to nano drug release research in wound healing, which were all co-cited no less than 50 times. The reference ranking first had been co-cited for164 times, while the remains were all co-cited less than 110 times. References with citation bursts are those papers that have been widely cited for some time. In CiteSpace, the k value for the g-index was set to 5, and the minimum duration of the burst was assigned to two years. We finally detected twenty-five references with intense citation bursts in the last twenty tears (Figure 8A). In Figure 8A, the period in which a reference was found to have a burst is displayed by a red line, indicating the beginning year and the ending year of the duration of the burst. As shown in Figure 8A, references with citation bursts first appeared in 2010, and the study that had the most potent burst is a paper published in 2011 [10]. Approximately 72.0% of the references had citation bursts between 2015 and 2020. The citation burst of the most recent reference appeared in 2017 [11]. We also used CiteSpace to create a network map of co-cited references. As shown in Figure 8B,C, all nodes representing the references which were classified into 13 specific clusters, including “#0 using nanotechnology”, “#1 healing diabetic wound”, “#2 excellent antibacterial activity”, and “#3 granular hydrogel”.

**Table 4:** Top 10 co-cited references related to nano-drug release in the wound healing field.

Rank	Co-Cited Reference	Co-Citation
1	Kamoun EA, 2017, J ADV RES, V8, P217, DOI 10.1016/j.jare.2017.01.005 [12]	83
2	Zhao X, 2017, BIOMATERIALS, V122, P34, DOI 10.1016/j.biomaterials.2017.01.011 [11]	75
3	Qu J, 2018, BIOMATERIALS, V183, P185, DOI 10.1016/j.biomaterials.2018.08.044 [13]	69
4	Liang YP, 2019, SMALL, V15, P0, DOI 10.1002/smll.201900046 [14]	59
5	Hamdan S, 2017, ACS CENTRAL SCI, V3, P163, DOI 10.1021/acscentsci.6b00371 [7]	55
6	Han G, 2017, ADV THER, V34, P599, DOI 10.1007/s12325-017-0478-y [1]	46
7	Zhao X, 2018, NAT COMMUN, V9, P0, DOI 10.1038/s41467-018-04998-9 [15]	45
8	Simoes D, 2018, EUR J PHARM BIOPHARM, V127, P130, DOI 10.1016/j.ejpb.2018.02.022 [16]	43
9	Mao CY, 2017, ACS NANO, V11, P9010, DOI 10.1021/acsnano.7b03513 [17]	37
10	Qu J, 2019, CHEM ENG J, V362, P548, DOI 10.1016/j.cej.2019.01.028 [18]	35

### 3.6. Keywords

**Figure 9:**
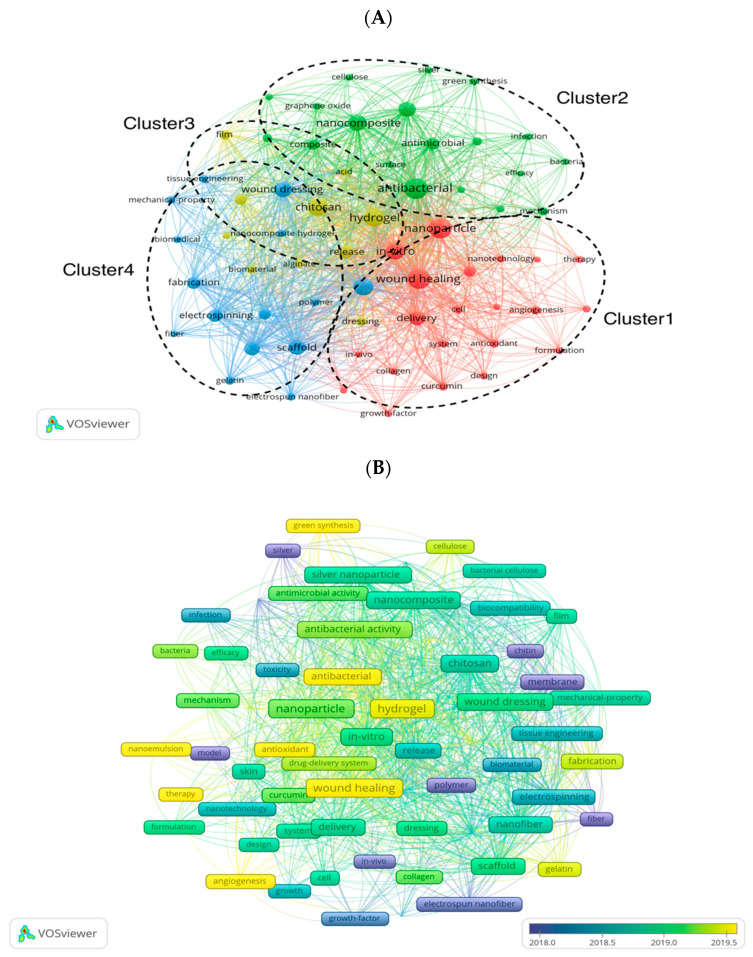

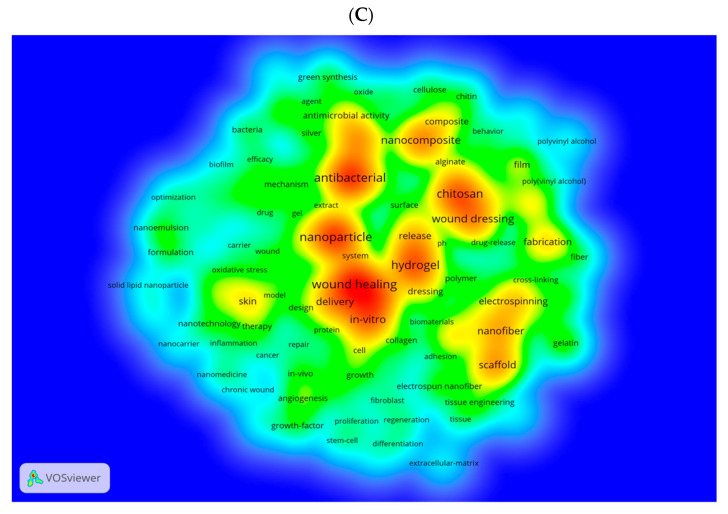
(**A**) The network map of keywords for NDSRSs research in the wound healing field generated by VOSviewer. (**B**) Overlay visualization of keywords generated by VOSviewer. (**C**) The density map of keywords for NDSRSs in the wound healing field was generated by VOSviewer.

Figure 9A displays a network map for keywords that appeared more than 50 times and were generated by VOSviewer. We extracted 6733 keywords from the 2076 articles and set the frequency of occurrence to at least 50. Finally, 65 keywords met the threshold and were included for analysis. And it can be seen that in the overlay visualization map of these 65 keywords (Figure 9B), the blue nodes represent keywords that appeared in the early stage. In contrast, the yellow ones represent the most recently appeared keywords. Instantly, keywords like “chitin”, “polymer”, and “electrospun nanofiber (NF)” were the main topics in the early stage, and keywords “wound healing”, “antibacterial”, “hydrogel”, “antioxidant”, and “angiogenesis” were hot topics in recent years. As shown in Figure 9C and Table 5, antibacterial was the most important keyword, with 539 (5.73%) co-occurrences, followed by wound healing (519, 5.52%), nanoparticle (516, 5.49%), hydrogel (429, 4.56%), chitosan (405, 4.31%), drug delivery (387, 4.12%), and in vitro (361, 3.84%). Among the top 20 keywords, some were related to types of nanomaterials, such as nanoparticles, nanofibers, and nanocomposites. Others were related to wound care, such as wound healing, dressing, and antibacterial. Some were relevant to drug releases, such as controlled release and drug delivery. Some polymers often used as nanocarriers were included in the 65 keywords, such as cellulose, alginate, collagen, and polyvinyl alcohol (PVA). Since they are often used in NDSRSs, these polymers might appear in the top 20 keywords in the future.

Clustering analysis was also performed for co-occurrence keywords, as shown in Figure 9A. Cluster 1 was the largest and contained 21 keywords, including wound healing, nanoparticle, growth factor, in vitro, antioxidant, angiogenesis, collagen, graphene oxide, curcumin, and skin. Cluster 2 contained 19 keywords, mainly related to antibacterial, antimicrobial, infection, nanocomposite, silver nanoparticle, gold nanoparticle, green synthesis, cellulose, surface, and mechanism. Cluster 3 comprised 15 keywords: wound dressing, drug delivery, controlled release, scaffold, nanofiber, fabrication, electrospinning, tissue engineering, biomedical, gelatin, etc. Cluster 4 contained 10 keywords: hydrogel, chitosan, acid, alginate, biomaterial, chitin, dressing, film, release, and membrane. Supplementary Figure S3 shows the top 25 keywords with the strongest citation burst. We can see that silk fibroin and graphene are the newly burst keywords, indicating that they might become the hot research materials for NDSRSs in wound healing. Moreover, keywords like chitin, biocompatibility, cytotoxicity, and membrane got their burst at a relatively early stage, and the keywords “fiber” has the most extended time of citation burst.

**Figure 10:**
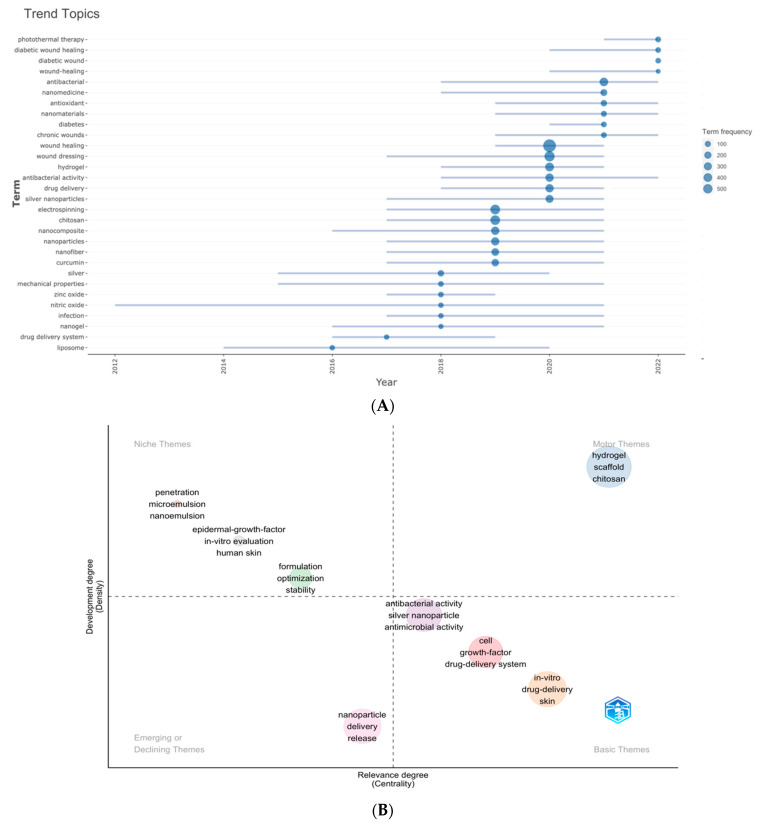
(**A**) Trend topics. The *X*-axis represents the year, while the *Y*-axis represents the cumulative keyword occurrences. (**B**) The keywords thematic map generated by R-Bibliometrix. The *X*-axis represents the centrality indicating the importance of a theme; The *Y*-axis symbolizes the density indicating the development of a theme.

Figure 10A is the trend topics map generated by the occurrence frequency of author keywords. We set the minimum frequency of words to 10 and displayed six words per year. The results showed that the duration of “nitric oxide” was the most extended (9 years), followed by “mechanical properties” (6 years). “Photothermal therapy” began to appear in the field of nano-based drug release for wound healing in 2021, and “diabetic wound healing” began to appear in 2020. “Antibacterial” had the highest frequency in 2021, while “photothermal therapy”, “diabetic wound healing”, and “diabetic wound” had the highest frequency in 2022. 

Figure 10B is the thematic keyword map conducted by R-Bibliometrix, we examined 260 keywords, where the minimum cluster frequency was six, and the number of labels for each cluster was 10. Only one cluster in the upper right quadrant (motor theme) has high density and centrality characteristics, representing well-developed and critical themes for structuring the NDSRSs for wound healing research. And the cluster consists of three topics: “hydrogel”, “scaffold”, and “chitosan.” There are three clusters in the upper-left quadrant (niche theme): cluster one contained “penetration”, “microemulsion”, and “nanoemulsion”; cluster two included “formulation”, “optimization”, and “stability”; and cluster three consisted of “epidermal growth factor”, “in vitro evaluation” and “human skin.” And the cluster in the third quadrant (emerging or declining theme) includes “nanoparticle”, “release”, and “delivery” as the major themes. Finally, the fourth quadrant (basic themes) contains three clusters: cluster one included “antibacterial activity”, “silver nanoparticle”, and “antimicrobial activity”; cluster two had “cell”, “growth factor”, and “drug delivery system”; and cluster three included “in vitro”, “drug delivery” and “skin”.

## 4. Discussion

In a paper, keywords can help us get the topic and theme of the research more accurately and quickly. It is almost the same in scientometrics, where the analysis of countries, institutions, and journals can reflect the output and collaboration messages and, at best, reflect a rough research direction. However, they are not enough for us to grasp the topics of research in a field, which the analysis of keywords can achieve. They can not only reflect the prevalence of themself by their frequencies but also, by forming different clusters, help us understand the composition and evolutionary trend of the primary topics [42]. Table 5 exhibits the top 20 keywords based on their frequencies, which mainly relate to molecular biology, biomedicine, surgery, and other fields. However, according to the keywords with high rankings, such as “nanocomposite”, “nanoparticle”, “chitosan”, “hydrogel”, “drug delivery”, and “nanofiber”, etc., we can infer that the application of new nanotechnology and novel supportive matrix in wound healing should be the most popular research directions in this field. Our study collected 65 keywords with a frequency of more than 50 times for co-occurrence analysis. As shown in Figure 9A, these 65 nodes formed a complex network. The size of nodes reflected the frequencies of keywords, which could indicate the mainly discussed topics in the NDSRSsin the wound healing field. In addition, these keywords formed 4 clusters with distinct topics. Trend topic map based on the frequency of author keywords shows that “antibacterial”, “chitosan”, “hydrogel”, “antioxidant”, and “electrospinning” are the hot topics of research in this field in recent years (Figure 10A). In addition, from the overlay visualization map of these keywords (Figure 9B), we can see that most of them appeared relatively early. Keywords like “polymer”, “chitin”, “membrane”, and “electrospun nanofiber”, etc., appeared before the year 2018. However, they have not been discussed too much in recent years, which means they are not hot topics in the latest studies.

Concerning the keywords thematic map generated by R-Bibliometrix (Figure 10B), there is only one cluster in the right upper quadrant, which includes “hydrogel”, “scaffold”, and “chitosan.” They are characterized by a high density and centrality, representing that they are well developed and are at the core position in the research of the nano-drug sustained release in wound healing. Keywords like “antibacterial activity”, “silver nanoparticle”, “growth factors”, and others located in the third quadrant are hot topics in current studies which are not developed well. It means that they will be discussed more in future research. 

Based on the analysis above, we knew that the frequencies of keywords only reflected the overall research level in this field from 1999–2022. However, most appeared relatively early, insufficient for us to grasp the frontier of research in this field. Technologies or methods highly studied in the latest time can reflect the change of research direction and the appearance of new research hotspots, which led us to pay attention to the burst items in this field. Figure S3 shows the top 25 keywords with strong burst citations; it can be seen that keywords like “polymer”, “membrane”, “fiber”, and “composite”, etc., were burst in the early years before 2016. In addition, “nanocarrier”, “graphene”, and “silk fibroin” these keywords have been popping up in recent years. “Silk fibroin” and “graphene” have been in a state of sudden citation burst from 2020 to 2022(with the strength of citation burst of 3.39 and 3.8, respectively), indicating that they will be the hot research topic in this field in the next few years. However, both the word “graphene” and “collagen”, as shown in Figure 9A, appear in the top 65 keywords selected according to frequency, while the word “silk fibroin” has a frequency of less than 50. Although “collagen” has been discussed much more than “silk fibroin” in this field, it doesn’t have an intense citation burst, while “silk fibroin” has had a strong citation burst most recently. Figure S4 shows the change in the annual frequency of these three keywords from 2016 to 2022. It can be seen that the frequency of gelatin has been significantly higher than that of collagen and silk fibroin in the past two years and shows an increasing trend, indicating that it will still be a hot topic of research in the future. Although the frequency of silk fibroin protein has been lower than that of collagen and gelatin, it has been increasing, which should attract more researchers’ attention.

As shown in Figure 8A, the latest burst references are Zhao X (2017), Ahmed EM (2015), Fan LH (2016), and Boateng J (2015), of which Boateng J (2015) had the strongest burst citation strength at 10.17. This article reviewed the advanced wound dressings used to treat chronic wounds, including amputations, diabetic wounds, leg ulcers, pressure sores, and surgical and traumatic wounds [38]. Patients with these wounds usually have low immunity and a high risk of infections and complications. Furthermore, the possible prospect of deep wound healing was discussed, containing some emerging physical approaches, such as hyperbaric oxygen, negative pressure wound therapy, and laser wound healing in routine clinical care. Zhao X’s paper in 2017 was the most recently published reference with strong citation bust in our study. They developed a set of injectable conductive self-healed hydrogels based on quaternized chitosan-g-polyaniline (QCSP) and benzaldehyde group functionalized poly (ethylene glycol)-co-poly (glycerol sebacate) (PEGS-FA) as antibacterial, antioxidative, and electroactive dressings for cutaneous wound healing.

Moreover, their team prepared a series of adhesive antioxidative hemostatic conductive hydrogels in 2019 [15]. The application of injectable nanocomposite conductive hydrogel dressings with multi-functions like adhesiveness, antibacterial activity, and suitable mechanical properties to enhance wound healing has been hotly discussed in this field in the last five years. Therefore, it will attract more and more attention from scholars in future research on NDSRSs for wound healing.

Nano-based drug sustained release systems have promising prospects in wound healing. According to the matrix structure of these carriers, they can be divided into liposomes, solid lipid nanoparticles (SLNs), nanostructured lipid carriers (NLCs), nanomicelles, nanofibers, nanoscaffolds, hydrogels, polymeric nanoparticles, etc. [8,43,44,45]. NDSRSs that combine nanotechnology with biomedicine have multiple advantages of increasing drug solubility, targeted drug delivery, transcellular drug delivery, and reducing drug side effects, showing obvious advantages in treating infections, local blood supply disorders, and metabolic disturbances in wound healing. Nevertheless, based on our study, nanocarriers and bioactive molecules of NDSRSs, including hydrogels, chitosan, silver nanoparticles, and growth factors, have been hotly discussed recently.

Hydrogels, which consist of different polymers, have been widely used in biomedicine because of their above 90% water content and excellent biocompatibility [46]. Hydrogels have been developed with nanotechnology. Nanohydrogels have more stimulation responses than conventional hydrogels. They can entrap active molecules with proper formation inside for better bioactivity, allowing them to be used as nanocarriers in drug-sustained release for wound healing [47,48]. Natural polymers, such as chitosan, cellulose, alginate, hyaluronic acid, collagen, and gelatin, have been applied to form nanohydrogels.

Among various natural hydrogels, chitosan, the product of deacetylated chitin, is the most frequently studied. In nanotechnology, chitosan is extensively used as a nano-scaffold material for tissue engineering. Its functional group, amino, contributes to forming adsorption-oriented nanofiber systems. Thus, it could combine with antimicrobial agents, metallic ions, growth factors, peptides, stem cells, and other drugs [49]. Jin Qu et al. [13] synthesized hybrid hydrogel mixed with quaternized chitosan for joint skin wound healing. The nano-system shows a faster wound healing rate in a full-thickness skin defect model. Clara López-Iglesias et al. [50] introduced chitosan aerogel—a new chitosan hydrogel loading vancomycin to treat chronic wounds. It is worth noting that chitosan has substantial antibacterial activities, making it an ideal nanocarrier for drug delivery in wound healing [16].

Because of its excellent properties, the nanocellulose-based composite has been widely used in biological fields like drug sustained release and wound healing. A large specific surface area and excellent mechanical properties can prolong the drug release time and improve the composite hydrogel’s physical strength [51]. For example, Congyang Mao et al. described a carboxymethyl cellulose (CMC) hydrogel encapsulating ZnO-Ag nanocomposite [17]. This nanoparticle delivery system significantly improved wound healing in a Wistar rats full-thickness wounds model. Bacterial cellulose (BNC) is a highly crystalline cellulose with a fine network structure formed by Acetobacter xylinus. Due to its high water-holding capacity, good biocompatibility, and tissue compatibility, BNC can be combined with a variety of antibacterial substances, such as various metal nanoparticles, forming the antibacterial nanocomposite dressing applied in the treatment of burns, scalds, chronic skin ulcers, and many other wounds [52].

Besides chitosan and cellulose, alginate, an anionic polysaccharide obtained from brown algae or bacteria, can also form a hydrogel by cross-linking. Alginate hydrogels share the properties of superior biocompatibility, low cost, and slow dissolution in wound fluids [53]. Furthermore, by crosslinking with divalent ions, alginate hydrogels can improve poor mechanical strength and biological stability [54]. In X. Li’s study, oxidized alginate was explored to prepare nanocomposite hydrogel to achieve a controlled release of curcumin [55]. Wang et al. developed calcium alginate-based hydrogel, which promoted a wound healing rate above 96%. Moreover, alginate hydrogels do not adhere to the wound site and can be easily removed without secondary damage [46,56].

Hyaluronic acid (HA) is a versatile polymer in fabricating HA-based products such as hydrogels, nanofibers, and 3D materials. Yongping Liang et al. obtained a HA/polydopamine-based nanocomposite with enhanced adhesion, hemostatic properties, and anti-oxidant abilities [14]. This nanocomposite promoted wound healing by significantly elevated vascularization, improved granulation tissue thickness, and collagen deposition in a mouse full-thickness wounds model. Moreover, CD44 is a receptor for HA, which is also found to be overexpressed in most cancer cells and associated with cancer progression. A study by F. Rosso et al. focused on the biodistribution of HA in a prostate cancer mice model and found a very low signal in healthy organs, whereas strong fluorescence in tumoral parenchyma. The results suggest potential HA-based biomaterials’ potential applications in cancer diagnosis and treatment [57].

Different synthetic polymers are also used to produce nanofibers, nanoparticles, and especially nanohydrogels, such as poly (lactic-co-glycolic acid) (PLGA), Polyvinylpyrrolidone(PVP), Polyvinyl alcohol (PVA) and poly(ε-caprolactone)(PCL). Among the numerous synthetic polymers, PLGA is one of the most commonly used to create nanocarriers. It is biodegradable and can achieve two-stage drug release when PLGA is combined with nanoparticlesz [58]. Furthermore, When PLGA is hydrolyzed, lactate is released from the polymer, promoting collagen synthesis and VEGF production [59]. However, in our study, PVA is the only synthetic polymer shown in the density map of keywords for NDSRSs. It is frequently employed in biomedical applications because PVA is biocompatible, hydrophilic, biodegradable, adhesive, and non-toxic. However, due to inert bioactivity and limited capacity for exudate absorption, PVA used alone as nanocarriers are insufficient to form functional wound dressing. Therefore, blending PVA with other bioactive molecules is essential to create superior NDSRSs [60]. Jin’s study found that sodium alginate/PVA composite showed optimal bio-adhesion and swelling properties compared with CMC, PVP, and PVA alone [61]. Nasef et al. [62] developed a novel PVA/chitosan/Ag nanocomposite hydrogel membrane that could keep a satisfactory swelling ratio and mechanical properties as elastic wound dressing while showing significant antibacterial ability against *Streptococcus mutans*.

Additionally, nanomicelles have shown promising prospects as nanocarriers for drug delivery because of their small size, outstanding biocompatibility, and ability to effectively encapsulate lipophilic drugs in their core [63,64,65]. Compared to conventional surfactant micelles, nanomicelles have lower dissociation kinetics after dilution by approximately 1000-fold, a superior advantage for drug delivery systems [66,67]. Zhiyong Zhang et al. [68] designed curcumin-alginate-based nanomicelles (C-A-NM) to treat colonic wounds. The results show that using C-A-NM can promote the healing of wounds in the gastrointestinal tract based on collagen induction and reduced bacterial activity. Moreover, the drug is embedded in the core by composition and modification of the polymer, reducing the irritation caused by direct contact between the drug and the skin, achieving the long-term therapeutic effect of the drug on the local wound, and reducing adverse reactions [69,70]. Various nanoemulsions have also been used as drug carriers. Oil-in-water (O/W) nanoemulsions are non-equilibrium, heterogeneous systems in which oil is the dispersed phase that is distributed into the continuous phase water and has the advantage of hydrophobic or lipophilic drug delivery. Pelin Secim-Karakaya et al. [71] developed functionalized cotton fabrics treated with topical O/W formulations containing bark extracts. After using the emulsion, the proliferation of Aspergillususion brasiliensis was significantly decreased. However, keratinocyte cell proliferation increases and the cell-free gap closure accelerates. An O/W nano emulsion carrier system presented by R. Vecchione et al. [72] exhibits a clear and significant time-dependent accumulation in tumor tissue and possible use as T2 weighed image contrast agent when the nanocomposite is loaded with cobalt ferrite oxide, which indicates a potential dual imaging use for carcinoma diagnosis. Furthermore, this O/W emulsion shows significant cytotoxicity in a murine melanoma model when the nanosystem is loaded with curcumin, thus showing a potentially therapeutic application.

For active bioactive molecules, silver nanoparticles and cell growth factors are hot topics in NDSRSs for wound healing. AgNPs are the most commonly used active antibacterial agent in nano-based drug delivery systems and have been widely applied in wound dressings [73], which is in accordance with the fact that antibacterial activity is a research hotspot of nano-based drug delivery for wound healing. Agnihotri’s study [74], AgNPs could inhibit bacteria such as *S. aureus*, *E. coli*, *B. subtilis*, *S. epidermidis*, and *Pseudomonas aeruginosa*. Yet, AgNPs could be more effective against Gram-negative bacteria. Silver ions are known for antibacterial properties, yet tissue toxicity has limited silver compounds’ application [75]. Therefore, different sizes and shapes of AgNPs were synthesized to overcome the drawbacks of silver compounds. It was demonstrated that AgNPs with high surface-to-volume could keep the effectiveness of the antibacterial activity with low concentration, which AgNPs further improved with the thiolated oligonucleotide [76,77]. Also, multiple new methods have been reported to keep the effectiveness of antimicrobial properties while decreasing the tissue toxicity of AgNPs, such as bacterial cellulose with AgNPs deposited or collagen-coated AgNPs encapsulated in collagen hydrogels [78,79]. Singla et al. synthesized the cellulose nanocrystals decorated with AgNPs [80]. The novel nanocomposites could optimize AgNPs concentration in wound dressing and increase essential growth factors while inhibiting inflammatory cytokines, including interleukin-6 and tumor necrosis factor-α in chronic wounds. Besides AgNPs, nanocrystalline silver is also considered helpful in wound surface area due to the sustained release of AgNPs. Commercial nanocrystalline silver wound dressings have already been developed. However, shortcomings of silver-based nanomaterials, such as the blue-coloration of the skin and the incidence of silver-resistant bacteria, still need to be addressed in the future [7]. It is also worth mentioning that curcumin, an anti-inflammatory molecule, is also a hot keyword in our study. In Abe and Huang’s respective studies, curcumin decreased the inflammatory cytokines secreted from macrophages and inflammation-related enzymes [81]. Merrell et al. explored curcumin-loaded nanofibers’ antioxidant and anti-inflammatory properties in a diabetic wound. Curcumin significantly reduced the level of interleukin-6 and exhibited a cytoprotective effect in the study [82].

Several growth factors (GFs), such as platelet-derived growth factor (PDGF), vascular endothelial growth factor (VEGF), fibroblast growth factor (FGF), transforming growth factor-β (TGF-β) and epidermal growth factor (EGF), are considered major bioactive molecules in the wound healing process [34,83]. Nevertheless, GFs are only effective in tropical administration. Wound exudates can block the biological effect of cell growth factors by inactivating GFs and preventing them from reaching wounds. Moreover, GFs are unstable in an open wound and quickly lose biological activity. On the other hand, nanotechnology could keep GFs from degradation more efficiently and release GFs in a sustained and controlled way [84,85]. Nanoparticles and nanofibers produced by electrospinning have been employed to carry GFs. Chereddy’s study [86] showed that PLGA/NPs containing VEGF promoted faster wound healing, which did not happen if VEGF alone was placed on the wound area. Chung’s study found that VEGF loaded in NPs in fibrin could be sustainedly released for more than four weeks. Bertoncelj [87] developed chitosan (CS)-based NFs loaded with platelet-rich plasma containing multiple GFs. The CS/NFs maintained the GFs’ metabolic activities in a moist environment. Z. Xie et al. [34] embedded nanoparticles with PDGF inside NFs to achieve sustained release of GFs. With the nanofibrous membranes, the nanostructure could release EGF for nine days after initial burst release and enhance collagen protein expression 25 times higher than normal cells [88]. Not only that, but there had also already been sequential GF-release nano-systems. Lai combined hyaluronic acid NFs, collagen NFs, gelatin NPs, and EGF gelatin NPs with different GFs.

The representative studies above inspired us to introduce nano-based drug release into wound healing treatment, which shows great potential in accelerating therapy. Figure 10A,B, analyzing the keyword trends in recent years, indicates that there are several hot research topics in nano-based wound healing, such as hydrogels, chitosan, scaffolds, and nanoparticles, which means that nanocarriers have recently become the research focus. Additionally, the trend topic analysis illustrates general directions for functional improvement of nano delivery systems, such as antibacterial, antioxidant, and controlled release. Generally, the analysis helps scholars to understand the current research frontier and provides a view of future exploration.

## 5. Conclusions

Our study conducted a systematic scientometric analysis of nano drug sustained release in wound healing between 1999 and 2022. In the last two decades, the number of publications related to nano drug sustained release in wound healing has increased rapidly, indicating that the interest of researchers in this field is increasing. It can be seen in the result that China has the most significant number of publications and citations, while the USA has the highest total link strength. Six of the top ten journals are from the Netherlands, and the top 10 institutions are from China or Iran. Islamic Azad Univ and INT J BIOL MACROMOL were the most productive institutions and journals, respectively. Jayakumar, R is the most influential author in this field. Based on the cluster analysis, trend topics, and thematic map, the hot topics in this area mainly focus on antibacterial, chitosan, scaffold, hydrogel, silver nanoparticle, and growth factors. In conclusion, this study provides a knowledge structure, evolution trend, and frontiers of research of nano drug sustained release in wound healing.

## Figures and Tables

**Table 1:** The top 10 journals and co-cited journals of nano-drug release research in the wound healing field.

Rank	Journal	N (%)	Country	IF (2021)	Co-Cited Journal	Co-Citations	Country	IF (2021)
1	INT J BIOL MACROMOL	121 (5.83%)	Netherlands	8.025	CARBOHYD POLYM	4560	Netherlands	10.732
2	CARBOHYD POLYM	59 (2.84%)	United Kingdom	10.732	BIOMATERIALS	4113	England	15.304
3	COLLOID SURFACE B	51 (2.46)	Netherlands	5.999	INT J BIOL MACROMOL	3575	Netherlands	8.457
4	INT J PHARMACEUT	48 (2.31%)	Netherlands	6.510	MAT SCI ENG C-MATER	2578	Netherlands	8.025
5	ACS APPL MATER INTER	45 (2.17%)	USA	10.383	ACS APPL MATER INTER	2350	USA	10.383
6	RSC ADV	39 (1.88%)	United Kingdom	4.036	INT J PHARMACEUT	2041	Netherlands	6.510
7	J DRUG DELIV SCI TEC	38 (1.83%)	France	5.062	ACTA BIOMATER	1802	England	10.633
8	Pharmaceutics	36 (1.73%)	Switzerland	6.525	J CONTROL RELEASE	1593	Netherlands	11.467
9	INT J NANOMED	35 (1.69%)	New Zealand	7.033	BIOMACROMOLECULES	1564	USA	6.979
10	Polymers	34 (1.64%)	Switzerland	4.967	ACS NANO	1532	Netherlands	18.027

**Table 2:** The top 10 countries and institutions contributed to publications of nano-drug release research in wound healing [n (%)].

Country	Rank	N (%)	Institute	N (%)	Country
1	CHINA	635 (30.59%)	Islamic Azad Univ	67 (3.23%)	Iran
2	INDIA	330 (15.90%)	Chinese Acad Sci	53 (2.55%)	China
3	IRAN	290 (13.97%)	Univ Tehran Med Sci	41 (1.97%)	Iran
4	USA	213 (10.26%)	Amirkabir Univ Technol	37 (1.78%)	Iran
5	SOUTH KOREA	125 (6.02%)	Sichuan Univ	35 (1.69%)	China
6	EGYPT	111 (5.35%)	Univ Tehran	34 (1.64%)	Iran
7	SAUDI ARABIA	75 (3.61%)	Natl Res Ctr	33 (1.59%)	China
8	ITALY	70 (3.37%)	Shanghai Jiao Tong Univ	32 (1.54%)	China
9	United Kingdom	61 (2.94%)	Isfahan Univ Med Sci	26 (1.25%)	Iran
10	BRAZIL	59 (2.84%)	Donghua Univ	22 (1.06%)	China

**Table 3:** The top 10 authors and co-cited authors of nano-drug release research in wound healing.

Rank	Author	N (%)	Citations	H-Index	Co-Cited Author	Citations	Total Link Strength
1	Jayakumar, R	14 (0.67%)	2545	46	Zhao, X	232 (0.42%)	1408
2	Sandri, Giuseppina	12 (0.59%)	497	40	Jayakumar, R	200 (0.36%)	788
3	Venkatasubbu, G D	12 (0.59%)	219	22	Liu, Y	178 (0.32%)	796
4	Han, Sung Soo	11 (0.53%)	211	34	Boateng, JS	175 (0.32%)	788
5	Guo, Baolin	10 (0.48%)	1640	65	Liang, YP	173 (0.32%)	1084
6	Alhakamy, Nabil A	10 (0.48%)	46	22	Li, Y	147 (0.27%)	593
7	Viseras, C	9 (0.43%)	451	31	Augustine, R	141 (0.26%)	719
8	Rossi, Silvia	9 (0.43%)	386	42	Archana, D	139 (0.25%)	646
9	Kim, Cheol S	9 (0.43%)	628	53	Li, J	136 (0.25%)	641
10	Parakash, J	(0.43%)	159	7	Kamoun, EA	135 (0.25%)	615

**Table 5:** The top 20 keywords in terms of frequency for NDSRSs in the wound healing field.

Rank	Keyword	N (%)	Rank	Keyword	N (%)
1	antibacterial	539 (5.73%)	11	silver nanoparticle	280 (2.98%)
2	wound healing	519 (5.52%)	12	delivery	252 (2.68%)
3	nanoparticle	516 (5.49%)	13	nanofiber	241 (2.56%)
4	hydrogel	429 (4.56%)	14	Electrospinning	204 (2.17%)
5	chitosan	405 (4.31%)	15	release	200 (2.13%)
6	drug delivery	387 (4.12%)	16	fabrication	180 (1.92%)
7	In vitro	361 (3.84%)	17	membrane	152 (1.62%)
8	nanocomposite	322 (3.43%)	18	controlled release	151 (1.61%)
9	wound dressing	303 (3.22%)	19	skin	150 (1.60%)
10	scaffold	285 (3.03%)	20	composite	129 (1.37%)

## Data Availability

The dataset can be downloaded from WOSCC according to the retrieval criteria mentioned in the methods.

## Supplementary Materials

The following supporting information can be downloaded at: https://www.mdpi.com/article/10.3390/pharmaceutics15041168/s1, Supplementary S1. Detailed search strategy; Supplementary Figure S1. The network visualization diagram of journal co-citation analysis. One node represents one journal, and the area means the citation frequency and the node size are reflected with co-citations. Data was processed using a VOS viewer. Supplementary Figure S2. The global distribution map of countries generated by R-Bibliometrix. Supplementary Figure S3. The top 25 keywords with the strongest citation bursts. Data was processed by CiteSpace. The time period in which a keyword was found to have a burst is displayed by a red line, indicating the beginning year and the ending year of the duration of the burst. Supplementary Figure S4. The annual frequency of “collagen”, gelatin”, and “silk fibroin” from 2016 to 2022.

## Abbreviations

The following abbreviations are used in this manuscript:

BC	Betweenness centrality
BNC	Bacterial cellulose
CMC	Carboxy methyl cellulose
C-A-NM	Curcumin-alginate-based nanomicelles
CNC	Cellulose nanocrystals
CNFs	Cellulose nanofibrils
CS	Chitosan
EGF	Epidermal growth factor
FGF	Fibroblast growth factor
GF	Growth factor
HA	Hyaluronic acid
NP	Nanoparticle
NF	Nanofiber
NDSRSs	Nano-based drug sustained release systems
PDGF	Platelet-derived growth factor
PLGA	Poly lactic-co-glycolic acid
PVP	Polyvinylpyrrolidone
PVA	Polyvinyl alcohol
PCL	Poly(ε-caprolactone)
PEGS-FA	Poly (ethylene glycol)-co-poly (glycerol sebacate)
QCS	Quaternized chitosan
QCSP	Quaternized chitosan-g-polyaniline
AgNP	Silver nanoparticle
SLNs	Solid lipid nanoparticles
NLCs	Nanostructured lipid carriers
TLS	Total link strength
TGF-β	Transforming growth factor-β
VEGF	Vascular endothelial growth factor
