# Supplementary material for: Scientometric Research on Trend Analysis of Nano-Based Sustained Drug Release Systems for Wound Healing
Source: Pharmaceutics. 2023 Apr 6;15(4):1168. doi: 10.3390/pharmaceutics15041168 (PMC10145462; doi:10.3390/pharmaceutics15041168)
Supplement: Supplementary file 1 [file pharmaceutics-15-01168-s001.zip › pharmaceutics-2247767-supplementary.pdf]

Supplementary

Supplementary S1. Detailed search strategy： #1 AND #2 AND #3

#1 ((((((((((ALL=(skin)) OR ALL=(cutaneous)) OR ALL=(wound)) OR ALL=(wound healing)) OR ALL=(wound repair)) OR ALL=(wound regeneration)) OR ALL=(wound closure)) OR ALL=(scar)) OR ALL=(burn)) OR ALL= (diabetic foot ulcer)) OR ALL=(surgical wound dehiscence)) OR ALL=(chronic wound)
#2 ((((((((((((((((((((((TS=(nanogels)) OR TS=(nanoemulsion)) OR TS=(nanoconjugate)) OR TS=(nanoliposome)) OR TS=(nanowire)) OR TS=(nanocarrier)) OR TS=(nanotech)) OR TS=(nanocluster)) OR TS=(nanodevice)) OR TS =(nanocomposite)) OR TS=(nanosheet)) OR TS=(nanorod)) OR TS=(nanotube)) OR TS=(nanofiber)) OR TS= (nanosphere)) OR TS=(quantum dot)) OR TS=(nanomaterial)) OR TS=(nanodot)) OR TS=(nanomedicine)) OR TS= (nanotechnology)) OR TS=(Nanocrystal)) OR TS=(Nanocrystalline Material)) OR TS=(nano particle)) OR TS= (nanoparticle)
#3 ((((((((((((((((((TS=(hydrogel)) OR TS=(liposomes)) OR TS=(dendrimers)) OR TS=(nisosomes)) OR TS= (nanocapsules)) OR TS=(drug carriers)) OR TS=(nanoemulsion)) OR TS=(hydrogel)) OR TS=(sustained release drug)) OR TS=(nanogels)) OR TS=(controlled release)) OR TS=(Delayed-Action Preparations)) OR TS=(Extracellular vesicle)) OR TS=(nanocomposite)) OR TS=(nanocomposite)) OR TS=(Exosome)) OR ALL=(micelles)) OR ALL= (colloids)) OR ALL=(dendrimers)) OR ALL=(emulsions)

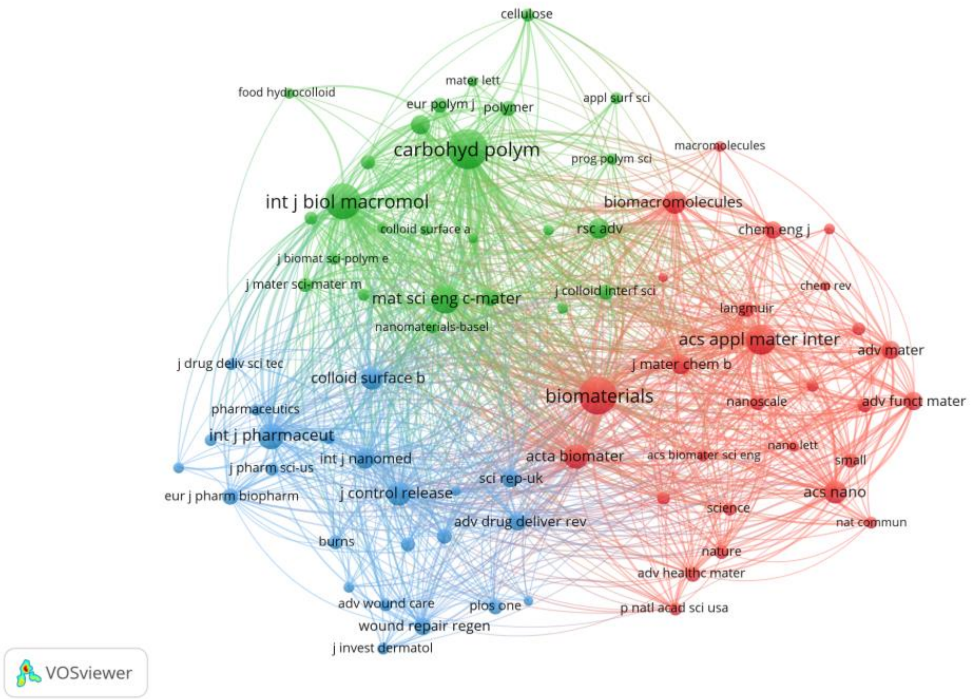

Supplementary Figure S1. The network visualization diagram of journal co-citation analysis. One node represents one journal, and the area means the citation frequency, the nodes size are reflected with co-citations. Data was processed using VOS viewer.

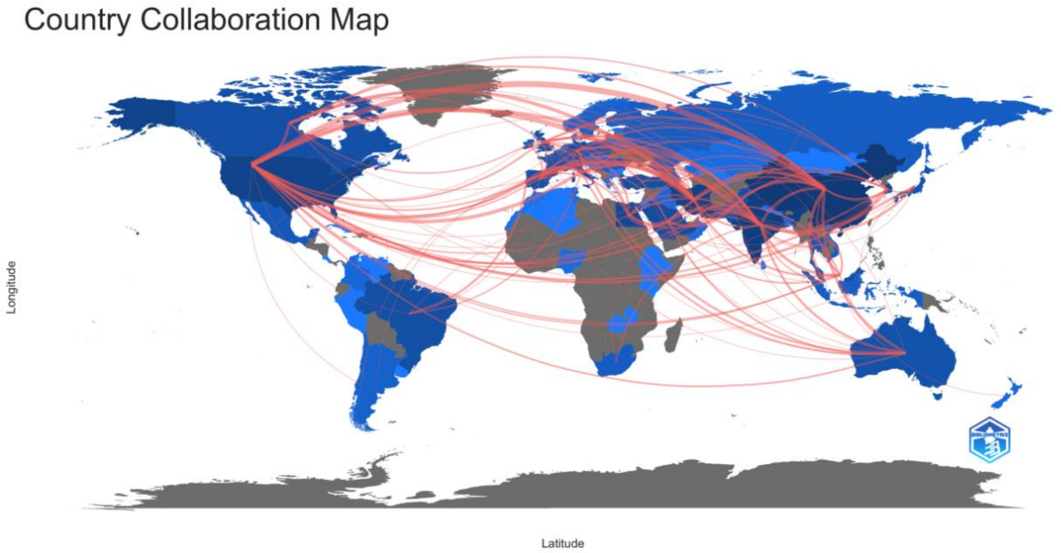

Supplementary Figure S2. The global distribution map of countries generated using R-Bibliometrix

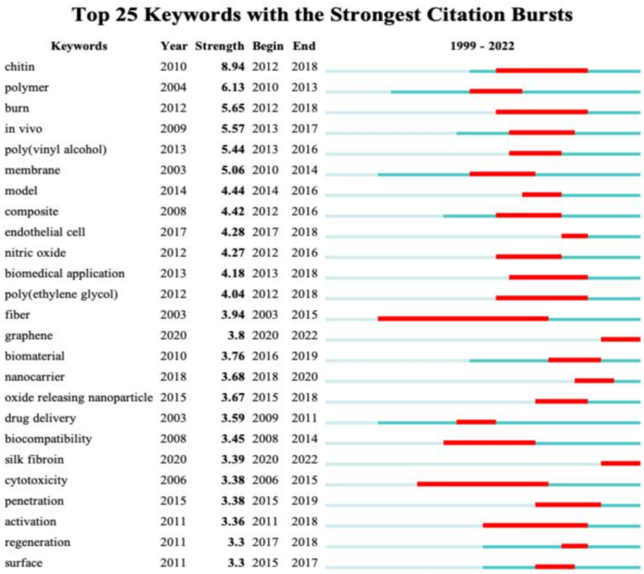

Supplementary Figure S3. The top 25 keywords with the strongest citation bursts. Data was processed using CiteSpace. The time period in which a keyword was found to have a burst is displayed by a red line, indicating the beginning year and the ending year of the duration of the burst.

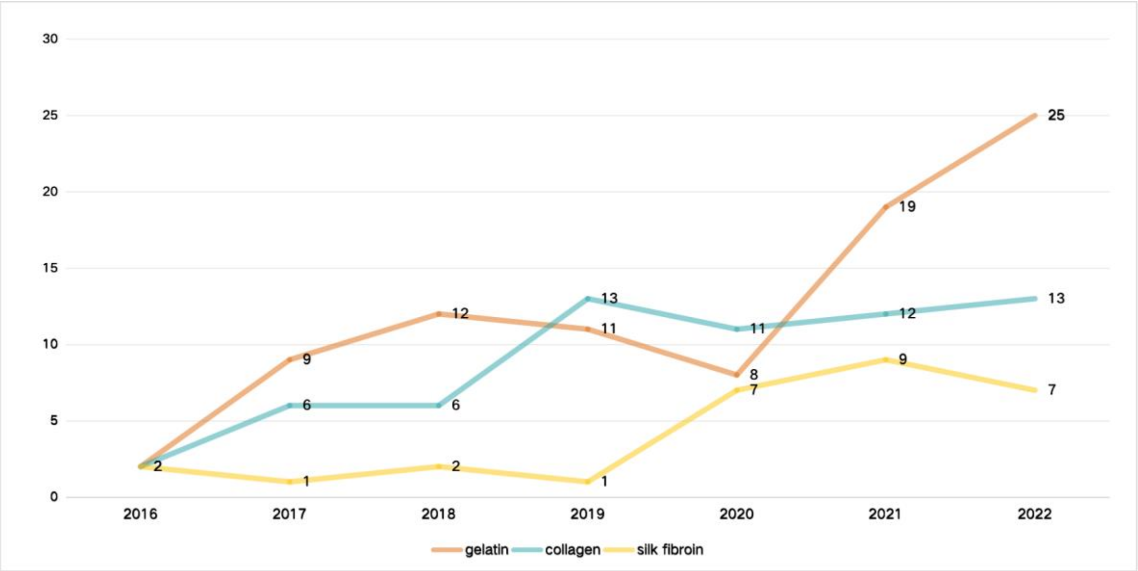

Supplementary Figure S4. The annual frequency of “collagen”,”gelatin”, and “silk fibroin” from 2016 to 2022
